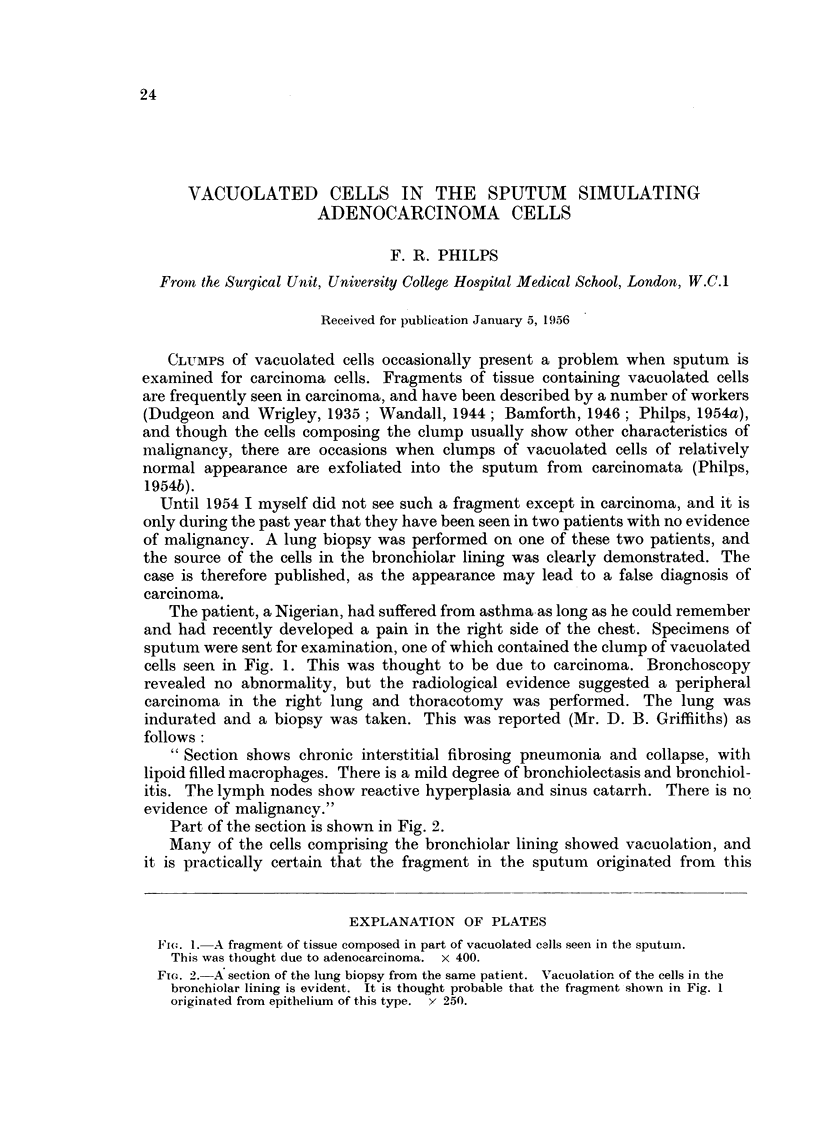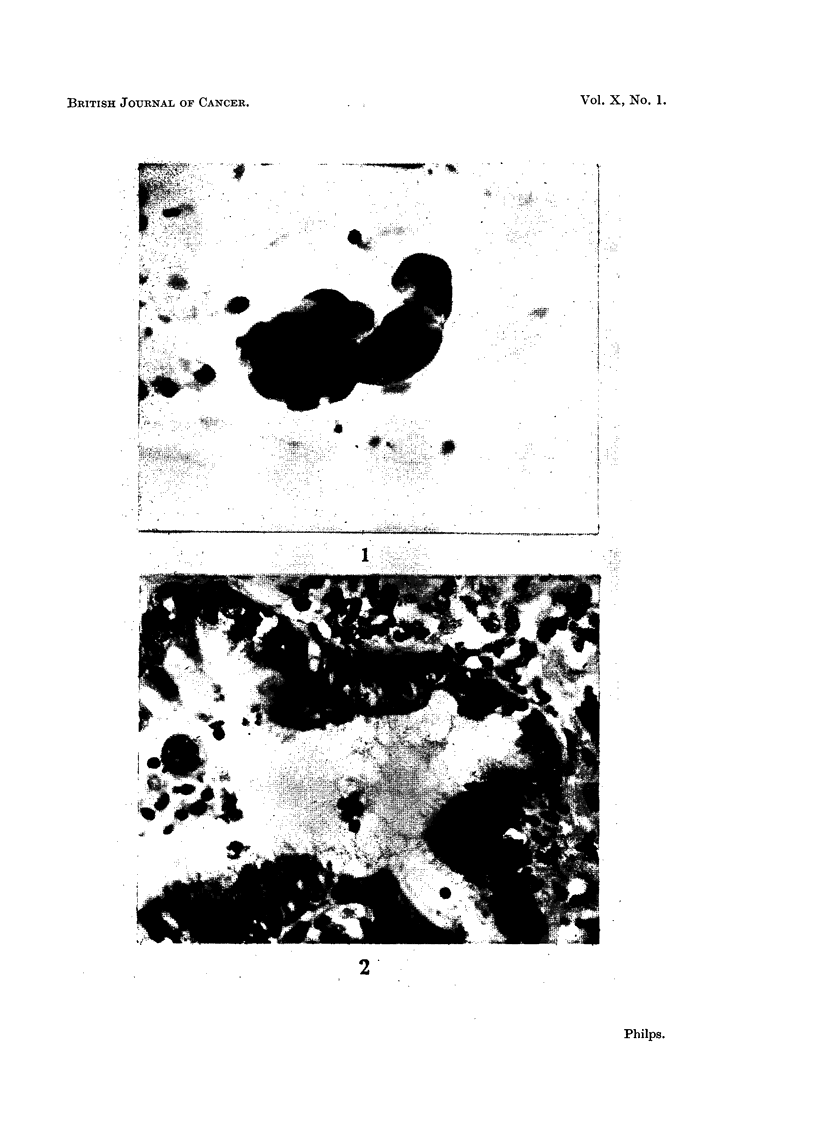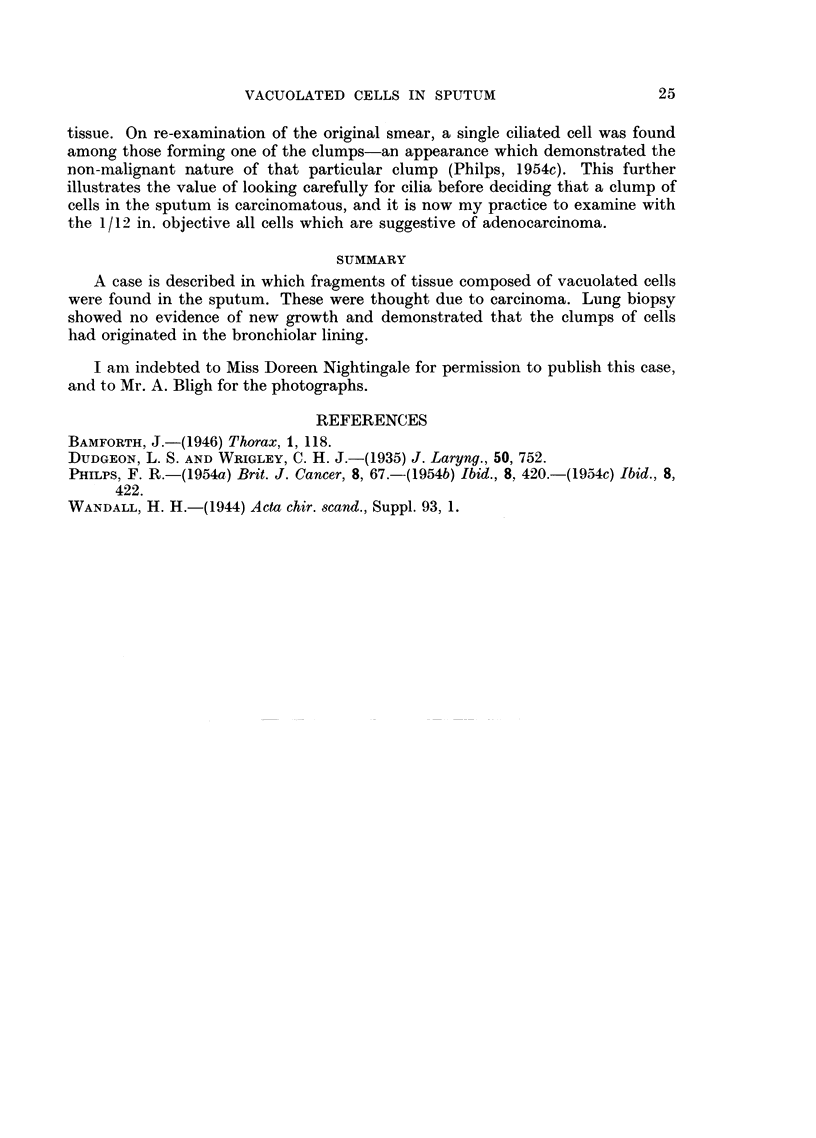# Vacuolated Cells in the Sputum Simulating Adenocarcinoma Cells

**DOI:** 10.1038/bjc.1956.3

**Published:** 1956-03

**Authors:** F. R. Philps

## Abstract

**Images:**


### VACUOLATED CELLS IN THE SPUTUM SIMULATING ADENOCARCINOMA CELLS

F. R. PHILPS

*From the Surgical Unit, University College Hospital Medical School, London, W.C.1*

Received for publication January 5, 1956

CLUMPS of vacuolated cells occasionally present a problem when sputum is examined for carcinoma cells. Fragments of tissue containing vacuolated cells are frequently seen in carcinoma, and have been described by a number of workers (Dudgeon and Wrigley, 1935; Wandall, 1944; Bamforth, 1946; Philps, 1954*a*), and though the cells composing the clump usually show other characteristics of malignancy, there are occasions when clumps of vacuolated cells of relatively normal appearance are exfoliated into the sputum from carcinomata (Philps, 1954*b*).

Until 1954 I myself did not see such a fragment except in carcinoma, and it is only during the past year that they have been seen in two patients with no evidence of malignancy. A lung biopsy was performed on one of these two patients, and the source of the cells in the bronchiolar lining was clearly demonstrated. The case is therefore published, as the appearance may lead to a false diagnosis of carcinoma.

The patient, a Nigerian, had suffered from asthma as long as he could remember and had recently developed a pain in the right side of the chest. Specimens of sputum were sent for examination, one of which contained the clump of vacuolated cells seen in Fig. 1. This was thought to be due to carcinoma. Bronchoscopy revealed no abnormality, but the radiological evidence suggested a peripheral carcinoma in the right lung and thoracotomy was performed. The lung was indurated and a biopsy was taken. This was reported (Mr. D. B. Griffiths) as follows:

" Section shows chronic interstitial fibrosing pneumonia and collapse, with lipoid filled macrophages. There is a mild degree of bronchiolectasis and bronchiolitis. The lymph nodes show reactive hyperplasia and sinus catarrh. There is no evidence of malignancy."

Part of the section is shown in Fig. 2.

Many of the cells comprising the bronchiolar lining showed vacuolation, and it is practically certain that the fragment in the sputum originated from this

---

EXPLANATION OF PLATES

FIG. 1.—A fragment of tissue composed in part of vacuolated cells seen in the sputum. This was thought due to adenocarcinoma. × 400.

FIG. 2.—A section of the lung biopsy from the same patient. Vacuolation of the cells in the bronchiolar lining is evident. It is thought probable that the fragment shown in Fig. 1 originated from epithelium of this type. × 250.

1

2

Philps.

tissue. On re-examination of the original smear, a single ciliated cell was found among those forming one of the clumps—an appearance which demonstrated the non-malignant nature of that particular clump (Philps, 1954*c*). This further illustrates the value of looking carefully for cilia before deciding that a clump of cells in the sputum is carcinomatous, and it is now my practice to examine with the 1/12 in. objective all cells which are suggestive of adenocarcinoma.

### SUMMARY

A case is described in which fragments of tissue composed of vacuolated cells were found in the sputum. These were thought due to carcinoma. Lung biopsy showed no evidence of new growth and demonstrated that the clumps of cells had originated in the bronchiolar lining.

I am indebted to Miss Doreen Nightingale for permission to publish this case, and to Mr. A. Bligh for the photographs.

### REFERENCES

BAMFORTH, J.—(1946) *Thorax*, **1**, 118.
DUDGEON, L. S. AND WRIGLEY, C. H. J.—(1935) *J. Laryng.*, **50**, 752.
PHILPS, F. R.—(1954*a*) *Brit. J. Cancer*, **8**, 67.—(1954*b*) *Ibid.*, **8**, 420.—(1954*c*) *Ibid.*, **8**, 422.
WANDALL, H. H.—(1944) *Acta chir. scand.*, Suppl. 93, 1.